# Hygroscopicity and Cloud Condensation Nuclei Activity of Fresh and Aged Biomass Burning Particles

**DOI:** 10.1021/acsestair.5c00331

**Published:** 2026-02-13

**Authors:** Bin Bai, Aishwarya Singh, Tianchang Xu, Christos Stamatis, Kezhou Lu, Nara Shin, Chase K. Glenn, Omar El Hajj, Kruthika V. Kumar, Anita Anosike, Muhammad Isa Abdurrahman, Sachin S. Gunthe, Joseph J. O’Brien, Gabriel Isaacman-VanWertz, Rawad Saleh, Nga L. Ng, Pengfei Liu

**Affiliations:** † 1372 School of Earth and Atmospheric Sciences, Georgia Institute of Technology, Atlanta, Georgia 30332, United States; ‡ 37268 EE Division, Department of Civil Engineering, Indian Institute of Technology Madras, Chennai 600036, India; § Centre for Atmospheric and Climate Sciences, Indian Institute of Technology Madras, Chennai 600036, India; ∥ School of Chemical and Biomolecular Engineering, Georgia Institute of Technology, Atlanta, Georgia 30332, United States; ⊥ Department of Civil and Environmental Engineering, Virginia Tech, Blacksburg, Virginia 24061, United States; # Department of Atmospheric and Oceanic Sciences, 8783 University of California, Los Angeles, California 90095, United States; ¶ School of Health Sciences, 311308 Purdue University, West Lafayette, Indiana 47907, United States; ∇ School of Environmental, Civil, Agricultural, and Mechanical Engineering, 1355 University of Georgia, Athens, Georgia 30602, United States; ○ 53777 Aerodyne Research, Billerica, Massachusetts 01821, United States; ⧫ USDA Forest Service Southern Research Station, Athens, Georgia 30602, United States; †† School of Civil and Environmental Engineering, Georgia Institute of Technology, Atlanta, Georgia 30332, United States

**Keywords:** biomass burning particles, organic aerosol, CCN activity, hygroscopicity, photochemical aging

## Abstract

Biomass burning (BB) is a major source of atmospheric particles and trace gases, influencing climate change, air quality, and human health. During the Georgia Wildland-Fire Simulation Experiment, we measured the hygroscopicity (κ) and size-resolved cloud condensation nuclei (CCN) activity of BB particles from controlled burns of fuel beds representative of three ecoregions in Georgia, United States. Primary BB particles were predominantly organic, and photooxidation in an oxidation flow reactor produced secondary organic aerosol (SOA) in a new nucleation mode while transforming primary organic aerosol (POA) into oxidized POA (OPOA) in the pre-existing accumulation mode. We measured hygroscopic growth from 20% to 90% relative humidity using a quartz crystal microbalance and assessed size-resolved CCN activity for particles from 30 to 350 nm at supersaturation between 0.13% and 0.99%. We found that the hygroscopicity parameter of OPOA (κ_OPOA_ = 0.10–0.19) was higher than that of POA (0.04–0.10), reflecting the influence of heterogeneous oxidation, whereas the hygroscopicity parameter of SOA (κ_SOA_ = 0.07–0.14) fell between the two. Both fresh and aged BB particles displayed size-dependent κ values and evidence of external mixing, likely because of complex emission characteristics of fuel beds and size-dependent deposition processes. Growth factor-derived and CCN-derived κ values were consistent when accounting for such heterogeneity. A strong positive correlation was found between the mass-averaged κ and O/C ratio, described by the regression κ = 0.31 ± 0.02­(O/C) – 0.05 ± 0.02, which broadly agrees with previous findings for a wide range of laboratory SOA and ambient oxidized organic aerosols. This suggests the potential applicability of a generalized hygroscopicity parameterization across organic aerosols within acceptable uncertainty. Our results highlight the role of BB particles as significant CCN sources during atmospheric aging and emphasize the importance of heterogeneous oxidation in physicochemical evolution of BB particles.

## Graphical abstract

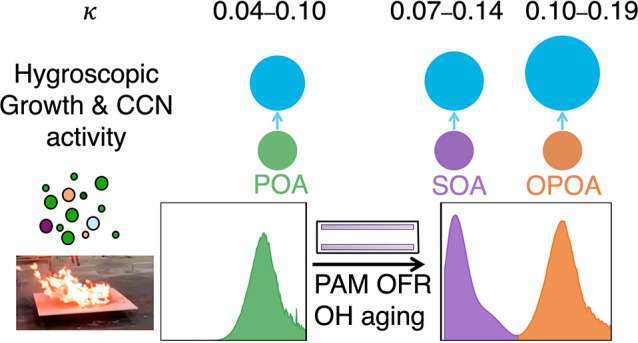

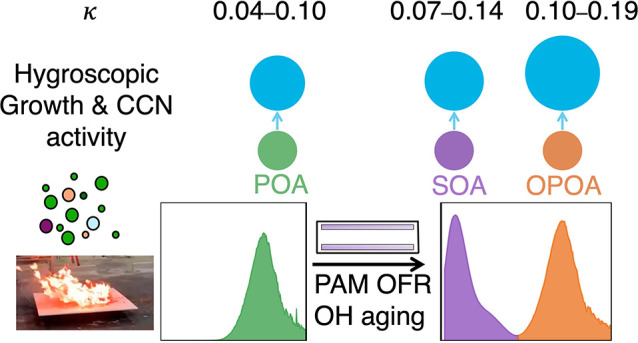

## 1 Introduction

Open land biomass burning (BB) is an important source of atmospheric aerosol particles.^1^ Wildfires are often ignited unintentionally or caused by natural processes.^2^ Prescribed fires are ignited intentionally with the purpose of forest management and are applied extensively in the Southeastern United States (U.S.).^3^ Both emit a great amount of BB particles and volatile organic compounds (VOCs). Under the effect of global warming, wildfires are experiencing significant increase in frequency, size, and intensity.^4,5^ The emitted BB particles can influence the climate directly through their interactions with light^6^ and indirectly via their role as cloud condensation nuclei (CCN) in supersaturation conditions (relative humidity, RH, above 100%).^7,8^ Previous studies have shown that the uncertainty associated with the CCN concentration from BB and its historical changes is one of the largest sources of uncertainty in the estimates of aerosol radiative forcing.^9–11^ When RH is below 100%, particle–water interactions and hygroscopic growth can occur, altering aerosol water content, visibility, and optical properties.^7^ Particles’ hygroscopic growth and CCN activation can be described by κ-Köhler theory, where a single hygroscopicity parameter κ is applied to represent the solute effect and the surface tension is assumed to be that of water.^12^

A major fraction of BB particles is organic, i.e., BB organic aerosols (BBOA). The relative proportion of organics and inorganics within BB particles^13–15^ and the properties of organic species^16,17^ play an important role in determining the κ values of BB particles. The κ values of BBOA also depend on fuels,^13,15,18^ burning conditions,^18,19^ and atmospheric aging.^14,19–24^ Bougiatioti et al.^22^ found that in the eastern Mediterranean, the κ value for freshly emitted BBOA was typically around 0.06, while atmospheric aged BBOA exhibited a higher κ value of 0.14. During atmospheric aging, BB primary organic aerosol (POA) can be oxidized by atmospheric oxidants through heterogeneous reactions, transforming into oxidized POA (OPOA). Oxidation of BB VOCs can produce species with low enough volatilities that form secondary organic aerosol (SOA) by condensing onto preexisting particles or nucleating new particles. Resolving the distinct physicochemical properties of OPOA and SOA helps understand the complex atmospheric evolution of BBOA. However, the hygroscopic growth of BBOA after aging and the effect of aging are often obscured by variable inorganic fractions in BB particles. Previous studies showed that the κ values of BB particles converged to 0.1–0.2 after aging, likely because the κ values of both SOA and OPOA fell within this range, while the relative abundances of highly hygroscopic inorganics and hydrophobic POA decreased after aging.^14,19^

The oxygen-to-carbon (O/C) ratios, commonly determined by aerosol mass spectrometer (AMS) measurements in laboratory and field studies,^25^ provide fundamental insights into the atmospheric oxidation of OA over time.^25–27^ Previous studies have reported a clear correlation between κ values and O/C ratios for laboratory-produced SOA^28–31^ and ambient oxidized organic aerosol (OOA).^28,32^ However, it remains unclear whether a similar correlation applies to atmospheric BBOA, considering the emission variability and atmospheric aging.

Hygroscopicity parameter κ for BB particles or BBOA has been determined using a humidified tandem differential mobility analyzer (HTDMA, reported as κ_HTDMA_) at subsaturation or using a cloud condensation nuclei counter (CCNC, reported as κ_CCN_) at supersaturation. Consistent κ_HTDMA_ and κ_CCN_ across subsaturation and supersaturation regimes were reported for BB particles in laboratory experiments^15,19,33^ and field measurements.^34^ In contrast, higher κ_CCN_ than κ_HTDMA_ values have been observed for water-soluble organic material of BB particles^35^ and SOA.^36^ The enhanced CCN activity therein was explained by gradual dissolution of less water-soluble species as water uptake increased.^35,36^ Although the solubility-limitation mechanism can reconcile the discrepancies between κ_CCN_ and κ_HTDMA_, the latter is typically measured at high RH ( ≥ 85%).^35,36^ The plausibility of this mechanism requires further validation using hygroscopicity data over a wider range of RH.^37^

The Georgia Wildland-Fire Simulation Experiment (G-WISE) aims to characterize the smoke emissions from wildfires and prescribed fires and to evaluate the effects of atmospheric photochemical aging.^38^ This study reports results focused on the hygroscopicity measurements of collected BB particles characterized by a quartz crystal microbalance (QCM) and in situ size-resolved CCN measurements. These measurements cover a wide range of RH from 20% to 90% and supersaturation (SS) from 0.13% to 0.99%, providing critical insights into the microphysical properties of both fresh and aged BB particles. Our results highlight dynamic and heterogeneous characteristics of BB particles as significant CCN sources and emphasize the importance of heterogeneous aging in their physicochemical evolution. We further examine the relationship between κ values and AMS-measured O/C ratios and propose a parameterization that broadly aligns with previous results for SOA and ambient OOA.

## 2 Materials and Methods

### 2.1 Burn Experiments

This study was performed as a part of the G-WISE. More detailed information on the burn experiments and procedures can be found elsewhere.^39^ A brief description is provided below.

#### 2.1.1 Collection of Fuel Samples and Fuel-Bed Preparation

In G-WISE, fuel beds were constructed to replicate the average proportions, mass loadings, and three-dimensional structures of fuel beds as observed in three ecoregions in Georgia. These ecoregions included the Oconee National Forest (Piedmont), Fort Stewart (Coastal Plain), and Chattahoochee National Forest in the southern Blue Ridge Mountains (Blue Ridge). The fuel beds consisted of recently senesced surface fuels, encompassing fine fuels (forest litter) and woody fuels (sticks and branches). Blue Ridge fuel beds also featured a layer of duff beneath the surface fuels. The fuel beds had an area of 0.5 m^2^, consistent with the scale of a “wildland fuel cell” unit.^40^ The moisture content of the fuel beds was conditioned to a dry (<4%) level to represent (drought-induced) wildfires or to a moist state to represent prescribed fires. For the prescribed burns, fine fuels were conditioned to a moisture content of 10–11%, and woody fuels were conditioned to 32–50% moisture levels.^41^ Notably, for the Blue Ridge fuel beds, duff ignition was excluded during prescribed fires because the high moisture content made the duff unavailable for combustion, whereas under wildfire conditions, the duff was sufficiently dry to burn. Overall, the experiments involved six burn permutations based on the combination of the ecoregion (Piedmont, Coastal Plain, and Blue Ridge) and burn condition (wildfire and prescribed fire), with duff combustion occurring exclusively in the Blue Ridge wildfire case.

#### 2.1.2 Experimental Procedures

The burns were conducted in a 1000 m^3^ burn room at the U.S. Forest Service’s Prescribed Fire Science Laboratory on the campus of the University of Georgia during October–November 2022. The burn room was equipped with an array of fans to attain well-mixed conditions. Sampling lines were routed from the burn room to an adjacent instrument room to perform both online measurements and offline sample collection. In a typical experiment, the burn was initiated in the morning with direct ignition. A radiometric thermal imager (Flir A655 sc), downsampled to 1 Hz thermography, was used to retrieve real-time combustion temperatures and to calculate the fire radiative power (FRP) throughout the burn. The burns typically concluded within 10 min as combustion temperatures fell below 573 K within all pixels. For experiments that involved duff ignition, the burn continued at low temperatures for approximately 60 min.^39^ By integrating FRP over the duration of the burn, we obtained the fire radiative energy (FRE, MJ). FRE normalized by the available fuel mass loading (FRE_norm_, MJ kg^–1^) was calculated for each burn. FRE_norm_ represents the efficiency with which fuel is converted to radiative energy and therefore serves as an indirect indicator of combustion efficiency and combustion conditions.

The smoke reached well-mixed conditions in the burn room within 10 min after the conclusion of the burn, and the highest aerosol mass concentrations were several mg m^–3^. After 30–60 min of filter collection, the burn room was vented for ∼30 min by rapidly bringing in fresh ambient air until the BB particle mass concentration was reduced to several hundred μg m^–3^ for photochemical aging and online aerosol and gas-phase measurements.

#### 2.1.3 Online Measurements and Plume Aging

The online measurements lasted for 4–5 h, during which the fresh particle mass concentration decreased with an e-folding lifetime of 2–4 h. By the end of each experiment, the particle mass concentration in the burn room remained much higher than that in the ambient background level. For fresh plume characterization, primary BB aerosol particles and vapors were directly measured by online instruments, including a high-resolution time-of-flight AMS (HR-ToF-AMS, Aerodyne Research), a scanning mobility particle sizer (SMPS 3082, TSI Inc.), a size-resolved CCNC (Droplet Measurement Technologies), and a Vocus-2R proton-transfer-reaction mass spectrometer (Vocus-2R PTR, Tofwerk AG.). All online measurements and offline sampling were conducted only after each burn had concluded, and the BB plume had become well mixed within the burn room. As a result, the reported aerosol properties represent the time-averaged characteristics of the emitted BB aerosols during the postcombustion dilution period, rather than the conditions associated with specific combustion phases.

To investigate the influence of photochemical aging, the primary BB aerosol particles and vapors were introduced into a PAM oxidation flow reactor (PAM OFR, Aerodyne Research) prior to online measurements. In the PAM OFR, two 185 nm ultraviolet lamps were used to initiate OH radical oxidation. The 185 nm radiation photolyzes O_2_ and H_2_O, producing O­(^1^D) that subsequently reacts with H_2_O to generate OH radicals.^29^ The total flow rate through the PAM OFR was 10 L min^–1^, corresponding to a residence time of 105 s. The experimental conditions alternated between “fresh” and “aged” every hour, allowing changes in particle chemical composition and physical properties during atmospheric aging to be characterized. Each experiment included two aged periods and two fresh periods, typically following an aged–fresh–aged–fresh sequence. A typical experimental sequence with the measured particle mass concentrations is shown in Figure S1. The lamp voltage was kept constant to maintain a stable aging condition, although the actual OH exposure (i.e., photochemical age) varied with the OH reactivity of the plume.

The OH exposure was determined from the decay ratios of Vocus-2R PTR-measured VOCs (toluene and benzene) during each “aged” period, as described by the following equation:

$$\ln\left(\frac{{\left[VOC\right]}_{aged}}{{\left[VOC\right]}_{fresh}}\right)=-k_{VOC+OH}\left[OH\right]t_{res}=-k_{VOC+OH}OHE$$

where [VOC]_fresh_ and [VOC]_aged_ are the concentrations of a specific VOC before and after passing through the PAM OFR, *k*_VOC+OH_ is the reaction rate constant between the VOC and OH, [OH] is the average OH radical concentration in the PAM OFR, *t*_res_ is the residence time in the reactor, and OHE represents the total OH exposure in the PAM OFR.

The OH exposure during the campaign ranged from 4.15 × 10^11^ to 9.64 × 10^11^ molecules cm^–3^ s, corresponding to 3.2–7.4 days of equivalent atmospheric aging, assuming an average ambient OH concentration of 1.5 × 10^6^ molecules cm^–3^. The average photochemical ages for all burns ranged from 5 to 6 days, except for the Blue Ridge wildfires, for which the photochemical ages in the PAM OFR were approximately 3 days, potentially due to the high OH reactivity (Table S1). A diagram illustrating the online measurements, offline collection, and PAM OFR aging is shown in Figure 1.

**Figure 1:**
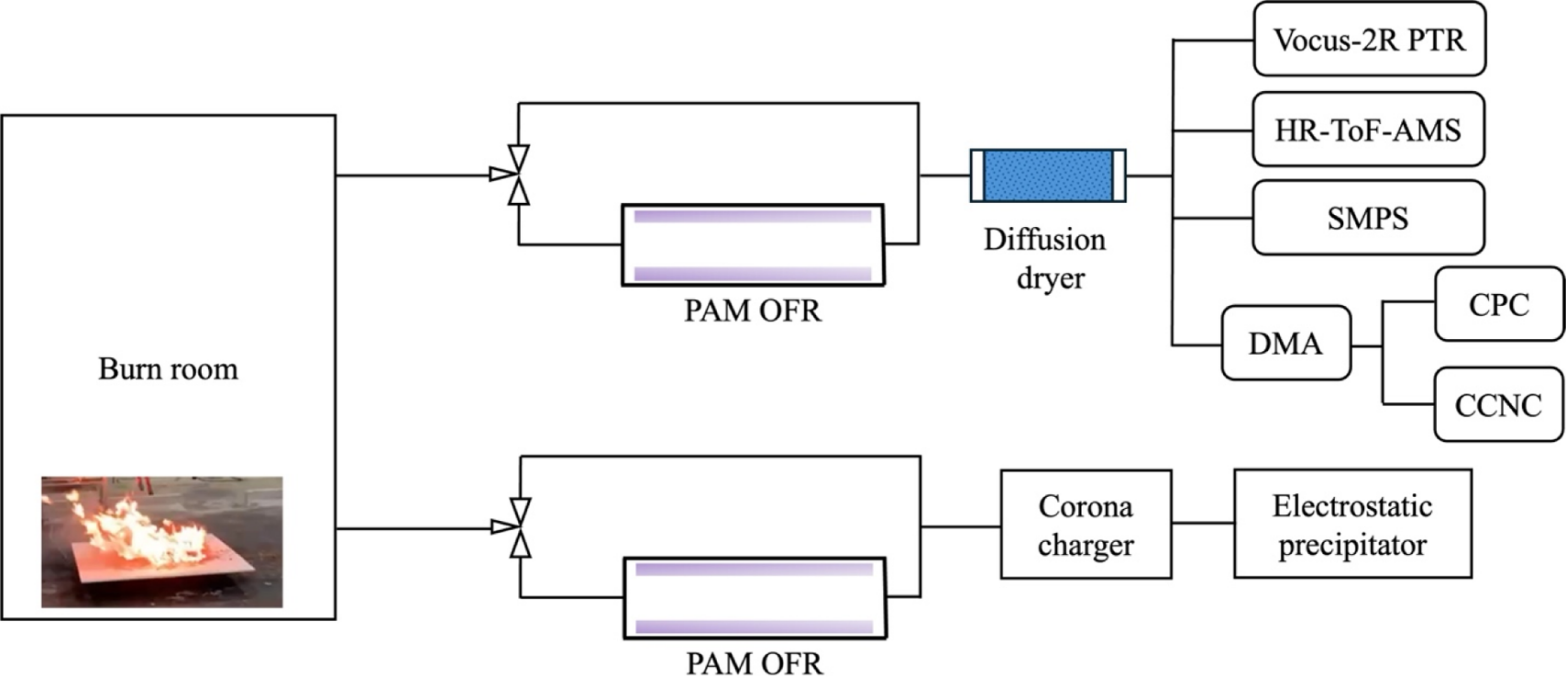
Schematic of the G-WISE burn experiments, online measurements, and offline sample collection.

### 2.2 QCM Sample Collection and Hygroscopicity Measurements

The fresh or aged BB particles were charged by a Corona Charger (IONER CC-8020) and then deposited onto a QCM quartz crystal (BL-QSX 303, nanoscience Instruments, SiO_2_-coated; or AT5-14-12-AU, Novaetech, gold-coated) using an electrostatic precipitator (TSI Nanometer Aerosol Sampler 3089). The collected samples were then stored at −18 °C until further analysis. The choice of substrate material did not significantly influence the hygroscopicity measurements (Figure S2). The collection efficiency, calculated using the SMPS-measured aerosol particle mass concentration, sampling flow rate, and collected particle mass, ranged from ∼10% to 50% (Table S2). The sampling time was between 5 and 400 min, depending on the BB particle concentrations. The prolonged sampling time did not systematically alter the chemical composition of BB particles as the variations in hygroscopicity across different sampling times were within the range of sample-to-sample variability (Figure S3).

For the hygroscopicity measurements, the particle-laden crystal sensors were mounted in a humidity- and temperature-controlled flow cell (Q-sense QFM401) that was purged with zero air at 30 cm^3^ min^–1^. The mass under different humidities was continuously monitored using a high-sensitivity QCM with dissipation (QCM-D, Q-sense Analyzer). The QCM operates on the principle that changes in the resonant frequency (Δ*f*) of the quartz crystal are proportional, through a sensitivity factor (ζ), to mass changes (Δ*m*) on the sensor. This relationship is expressed by the equation Δ*m* = −ζΔ*f*. To ensure accuracy, six different frequency overtones were cross-verified. The baseline of a blank sensor could be restored with an absolute mass error of <0.75 μg by cleaning the sensor with methanol and deionized water after each experiment, and this error was included in the overall uncertainty. Information on the collected particle masses for each sample is outlined in Table S2.

RH in the flow cell was switched between dry (<1% RH) and wet (20–90% RH) conditions every 20 min by adjusting the mixing ratio of dry and humidified zero-air flows using two mass flow controllers. Equilibrium was reached within 1 min after each RH switch, as indicated by the water uptake kinetics measured by the QCM,^42^ and the data after reaching the equilibrium state were used to calculate hygroscopicity (see Figure S4). RH was monitored by a RH sensor (Rotronic, HC2A-S) throughout the entire measurement. A schematic diagram of the QCM measurement setup can be found in our previous publication.^37,43^ The particle mass on the QCM sensor under dry conditions (*m*_dry particles_) was measured before and after each elevated-RH period to account for possible evaporation. The mass sensitivity of the QCM is <1 ng cm^–2^, corresponding to one-tenth of a single molecule layer of water.^37^ This sensitivity is sufficient for accurate detection of water uptake at RH > 20%. Surface adsorption, characterized by water uptake on a clean sensor, was measured and corrected. For SiO_2_-coated sensors, the adsorbed water mass (ng) was determined to be 0.25 × RH (%), and for gold-coated sensors, the adsorbed water mass (ng) was 0.10 × RH (%), based on clean-sensor measurements. Surface adsorption typically accounted for <5% of the total water uptake. The particle density (ρ_dry particle_) was estimated following the method of Kostenidou et al.^44^ by combining HR-ToF-AMS and SMPS size-distribution measurements. The volume-based hygroscopicity growth factor was then calculated, and the hygroscopicity parameter κ_QCM_ was calculated from the following equation:^12^

$$\kappa_{QCM}=\left(\frac{100}{RH\;\left(\%\right)}-1\right)\frac{m_{water}\rho_{dry\;particle}}{\rho_{water}m_{dry\;particle}}$$

where RH is the relative humidity during the humidified period, *m*_water_ is the mass of water absorbed by the particle film under humidified conditions, *m*_dry particle_ is the interpolated dry particle mass corresponding to the same humidified period, and ρ_water_ and ρ_dry particle_ denote the densities of water and BB particles, respectively. Because the thin film is macroscopically flat relative to particle size, the curvature effects are negligible, and the measured RH equals the water activity.

The QCM method was validated in our previous study^37^ using amorphous sucrose, α-pinene ozonolysis SOA, limonene ozonolysis SOA, toluene photooxidation SOA, and dodecane photooxidation SOA thin films across a wide range of RH, and showed good agreement with results obtained from other methods. The QCM method, due to its high collection efficiency, yields a mass- or volume-averaged hygroscopicity that reflects the behavior of larger particle sizes (the mode diameter in the volume-diameter distribution is ∼500 nm for the investigated BB particles here). As a result, κ_QCM_ should be more comparable with κ_CCN_ estimated at low SS with larger activation diameters. At the same time, this mass-averaged κ_QCM_ is more directly related to the bulk chemical composition measurements by HR-ToF-AMS, which is an advantage over traditional hygroscopicity measurements that typically probe only discrete particle diameters.

### 2.3 Size-Resolved CCN and κ_CCN_ Retrieval

Fresh or aged BB particles from the burn room were size-selected by a differential mobility analyzer (DMA, TSI 3082). The particles were dried to RH < 30% using a homemade diffusion dryer before entering the DMA. The CCN activation ratios were determined as a function of particle dry mobility diameter in diameter-scanning mode from simultaneous measurements of a continuous-flow CCNC and a condensation particle counter (CPC, TSI 3772). The SS levels in the CCNC were varied between 0.13% and 0.99%. Calibration of the CCNC SS was conducted before and after the campaign using ammonium sulfate particles. The detailed protocol for size-resolved CCNC calibration and measurement followed Rose et al.^45^ The doubly charged particle fraction was subtracted based on the particle size distribution and charging efficiency, and the DMA transfer function, calculated from the flow rate and DMA geometry, was corrected prior to data fitting.^46^ The spread of the corrected activation curve primarily reflects the heterogeneity in the mixing state of the particles.

The critical dry diameter, or activation diameter, which is the dry particle diameter at which 50% of the aerosol particles activate into cloud droplets for each SS, was determined by fitting the activation curve to a Gaussian error function. When two-stage activation was observed, we applied a weighted sum of two error functions, reflecting two externally mixed particle populations. We note that the CCN activation curve represents a continuous distribution of κ values, and the applied fittings should be interpreted as approximate bounding values between two apparent activation diameters.^15^

Each complete CCN measurement required one hour and consisted of six full diameter scans from 30 to 350 nm to determine the critical diameters at six different SS levels. Comparisons of CCN results from two “fresh” or two “aged” periods with different concentrations due to dilution showed good consistency, indicating that temporal variations introduced by dilution and potential dark aging were minor. This suggests that the observed SS- and diameter-dependent CCN activity reflects intrinsic aerosol properties rather than temporal artifacts.

## 3 Results

### 3.1 Hygroscopic Growth for Fresh and Aged BB Particles

Figure 2a–c shows the volume-based hygroscopic growth, defined as the volume ratio of the film at an elevated RH to that at <1% RH, for both fresh and aged BB particles from six different burn experiments. The particle film volumes increased continuously from 20% to 90% RH because of absorptive water uptake. No deliquescence behavior was observed for either fresh or aged BB particles. Continuous water absorption was also reported for BB particles from the burning of southwestern U.S. biomass fuels,^18^ as well as for particles emitted from most of the biomass fuels measured by Carrico et al.^13^ However, some samples from palmetto burns were inorganic-dominant and underwent deliquescence.^13^ The different hygroscopic growth behaviors observed across studies can be attributed to the variability in the organic and inorganic fractions of the BB particles. The deliquescence RH for mixtures of KCl-levoglucosan and (NH_4_)_2_SO_4_-levoglucosan was observed to decrease and ultimately disappear as the organic mass fraction increased.^16,17^ The hygroscopic growth of both fresh and aged BB particles observed in this study resembled that of SOA or of organic compounds that adopt an amorphous state^37,47^ because the nonrefractory portion of both fresh and aged BB particles was composed predominantly of organics (97–98%), as measured by HR-ToF-AMS (Table S3). Previous studies have reported that the organic fraction of primary BB particles ranges from 22% to 99% (Table 1), likely depending on the inorganic ion content of the fuels.^18^

**Figure 2:**
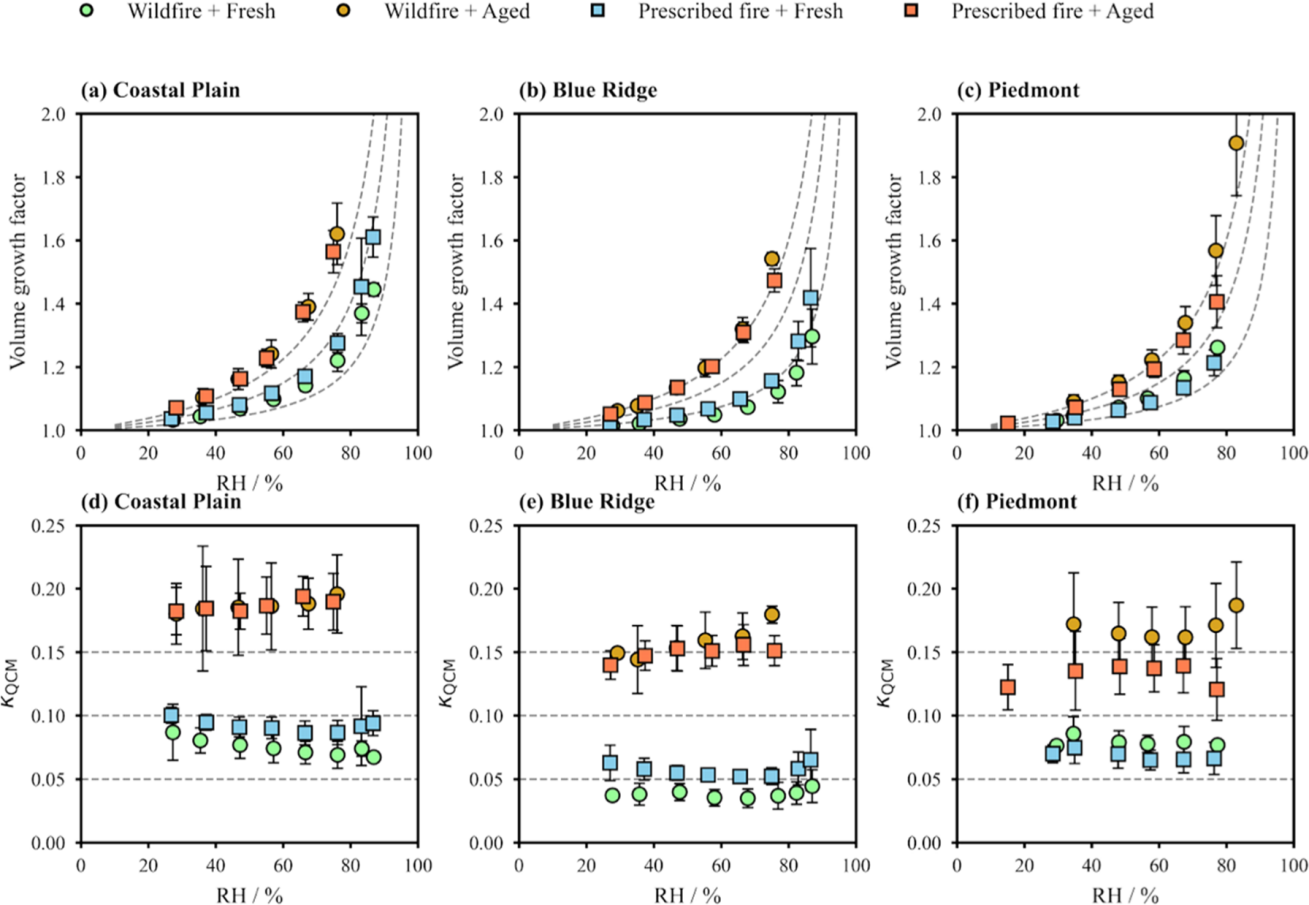
Volume growth factor (panels a–c) and hygroscopicity parameter (κ_QCM_, panels d–f) determined by QCM hygroscopicity measurements.

**Table 1:** Summary of Biomes, Instrumentation, and Hygroscopicity Parameters Reported in the Literature

	biomes	instrument	κ_fresh_	κ_aged_	κ_POA_	κ_OPOA_	κ_SOA_	OA fraction
this study	reconstructed fuel beds in Georgia, the U.S.	QCM	0.04–0.10	0.14–0.20	0.04–0.10			
		CCNC	0.06–0.10	0.07–0.19	0.06–0.10	0.10–0.19	0.07–0.14	
Petters et al.^15^	24 dry biomass fuels from the U.S. and Asia	HTDMA	0.02–0.8		0.05–0.19^a^			0.22–0.95 ^59^
		CCNC						
Carrico et al.^13^	33 dry biomass fuels from the U.S. and Asia	HTDMA	0.02–0.55		0.02–0.08^a^			0.43–0.99
Dusek et al.^33^	various wood	CCNC	0.05–0.082					
		HTDMA	0.037–0.061					
Engelhart et al.^14^	12 biomass fuels from North America	CCNC	0.06–0.6	0.08–0.3	0.06–0.10^a^		0.10 ± 0.02	0.22–0.95
Martin et al.^19^	beech log wood	CCNC	0.03–0.39	0.06–0.21^b^			0.07–0.10^c^	∼89%
		HTDMA	0–0.39	0.02–0.25^b^			0.03–0.06^c^	
Li et al.^60^	dried agricultural residues	HTDMA	0.19–0.32		0.087^c^			89%
Chen et al.^61^	dried peat, fern, and acacia	HTDMA	0.02–0.09					99%
Gomez et al.^18^	southwestern U.S. fuels	nephelometer	0–0.18					>75%
Li et al.^21^	dried saw grass	CCNC	0.17–0.24	0.23–0.34				>65%
Chen et al.^20^	dried peat, fern, and acacia	HTDMA	0.06–0.09		0.06–0.09^a^	0.10–0.32	0.09–0.23^d^	>96%
Mouton et al.^62^	dried Eucalyptus and cow dung	CCNC	0.021–0.577					
		CRDS^e^	0.012–0.3					

a Organic-carbon-dominant BB particles.

b Value derived after 6 h of aging.

c Value derived from composition-wise regression.

d Potential influence from sulfate.

e Cavity ring-down spectrometer.

For fresh BB particles emitted from prescribed fires and wildfires, the differences in the observed volume growth factors were minor compared to the effect of aging. Photochemical aging resulted in a 2–3-fold increase in the hygroscopic growth factor. Figure 2d–f shows the hygroscopicity parameters κ_QCM_ derived from hygroscopic growth, which remained constant across the investigated RH range of 20–90%. For fresh BB particles, the averaged κ_QCM_ ranged from 0.04 to 0.10, and the differences between particles from wildfires and prescribed fires were <0.02. Across all burns, we observed a weak positive relationship between FRE_norm_ and both the O/C ratios and the hygroscopicity of fresh BB particles (Figure S5). This result is consistent with previous work, demonstrating that FRE_norm_ is negatively associated with water-insoluble brown carbon fraction and may serve as a primary driver of differences in the physicochemical properties of primary BB particles.^39^

The measured κ_QCM_ values are consistent with previous studies reporting κ between 0.02 and 0.19 for organic-dominated BB aerosols or BB POA (Table 1). Carrico et al.^13^ reported that BB particles produced from the combustion of western U.S. montane, northwestern U.S., and Alaskan boreal fuels (e.g., pines, firs, duffs, and spruces) exhibited relatively low hygroscopicity (0.1), whereas those emitted from the burning of Asian rice straw, sugar cane, western U.S. rangeland sagebrush, and southeastern U.S. Coastal Plain palmetto and black needle rush were highly hygroscopic (κ > 0.4). This distinction was mainly driven by variations in the inorganic fraction.^13^ In this study, the fuel beds were reconstructed from a mixture of different fuel types, which yielded BB particles with compositions more convergent than those produced from single-fuel burns. The fuel beds representing the Coastal Plain, Blue Ridge, and Piedmont regions predominantly released organics and produced BB particles that fell into the least hygroscopic category identified by Carrico et al.^13^

After photochemical aging in the PAM OFR, κ_QCM_ increased notably to 0.14–0.20. The BB particle O/C ratios were also significantly elevated (Tables S1 and S3). The magnitude of κ_QCM_ enhancement (0.06–0.12) varied among burns and showed no clear correlation with photochemical age or with the corresponding increases in O/C ratios (Table S1). This suggests that the observed κ_QCM_ enhancement may primarily reflect intrinsic property differences in the properties of BB particles emitted from each burn.

Fresh, organic-dominant BB particles exhibit higher hygroscopicity than oxidized anthropogenic POA (APOA) such as bis­(2-ethylhexyl)­sebacate and lubricating oil,^29^ and their hygroscopicity further increases upon atmospheric aging. These results suggest that BB particles are capable of acting as CCN at relatively high supersaturations upon emission and can exert broader climatic impacts during atmospheric transport.

We did not detect any gradual dissolution or κ enhancement at elevated RH levels for fresh or aged BB particles. Given that the maximum volume growth factor observed was approximately 2, the hygroscopic growth beyond the 90% RH limit remained uncertain. We cannot rule out the possibility of enhanced κ at very high RH values, and future investigations should consider extending the RH range to higher values. Previous studies have examined the RH-dependent hygroscopicity of various types of SOA, where minor or even negative RH dependences were reported at low RH.^37,48,49^ For less-oxidized monoterpene SOA, discrepancies between subsaturation and supersaturation hygroscopicity have been attributed to the nonideality due to strong interactions between hydrophobic and hydrophilic species, as well as the surface tension reduction due to liquid–liquid phase separation at RH > 95%.^37^

### 3.2 Size-Resolved CCN Activity for Fresh and Aged BB Particles

The CCN activation curves at various SS levels for both fresh and aged BB particles are shown in Figure S6. At the lowest SS of 0.13%, the activation ratio remained below unity up to 350 nm for both fresh and aged BB particles. At higher SS (0.20–0.99%), complete activation was observed for both fresh and aged BB particles with diameters exceeding 250 nm, consistent with field observations.^24^ Specifically, BB particles showed a distinct two-stage activation pattern indicative of external mixing at SS = 0.20% for particles in the 100–300 nm size range (Figure S6 and Table S4). Several previous laboratory studies have also reported externally mixed BB particles emitted throughout burns.^13,15^ Since the measured BB particles represent a mixture from the full combustion emissions of reconstructed fuel beds, the emergence of externally mixed populations at larger diameters possibly reflects the complexity of fuel compositions and burning conditions.

The derived κ_CCN_ values are shown as a function of SS in Figure 3a–d and as a function of activation diameter in Figure 3e–h. The κ_QCM_ values (squares in Figure 3a–d) derived from subsaturation hygroscopic growth measurements are also shown for comparison. For fresh particles, the κ_CCN_ values derived at small activation diameters (<100 nm) were removed to avoid potential interference from ambient particles because the number concentrations of fresh BB particles were not significantly higher than those of ambient particles in this diameter range (Figure S7). The fresh BB particles from Coastal Plain wildfires, Coastal Plain prescribed fires, and Blue Ridge prescribed fires exhibited relatively constant κ_CCN_ values that were consistent with the corresponding κ_QCM_ values (κ_CCN_ = 0.087 ± 0.024, 0.080 ± 0.013, 0.072 ± 0.013, and κ_QCM_ = 0.075 ± 0.013, 0.092 ± 0.014, 0.057 ± 0.013, respectively). For particles emitted from Blue Ridge wildfires, κ_CCN_ (0.061 ± 0.009) was higher than the corresponding κ_QCM_ (0.038 ± 0.009). In Blue Ridge wildfires, emissions originated from both surface fuels and duff, in contrast to the rest of the permutations, where emissions were from only surface fuels. In addition, Blue Ridge wildfires exhibited a longer combustion duration at comparatively lower temperatures. Therefore, the Blue Ridge wildfire particles likely exhibited stronger emission heterogeneity because of more complex combustion conditions, and the observed discrepancy between κ_CCN_ and κ_QCM_ may be attributed to size-dependent variations in chemical composition across larger particle sizes. Hydrophobic species may preferentially condense onto larger particles during combustion, as suggested by the external mixing observed between 100 and 300 nm. Emission heterogeneity in Blue Ridge wildfires was also evident from their distinctive fresh particle size distributions, which displayed a clear multimodal pattern, whereas particles from other burns exhibited a single accumulation mode centered around 200 nm (green dashed lines in Figure 3e–h).

**Figure 3:**
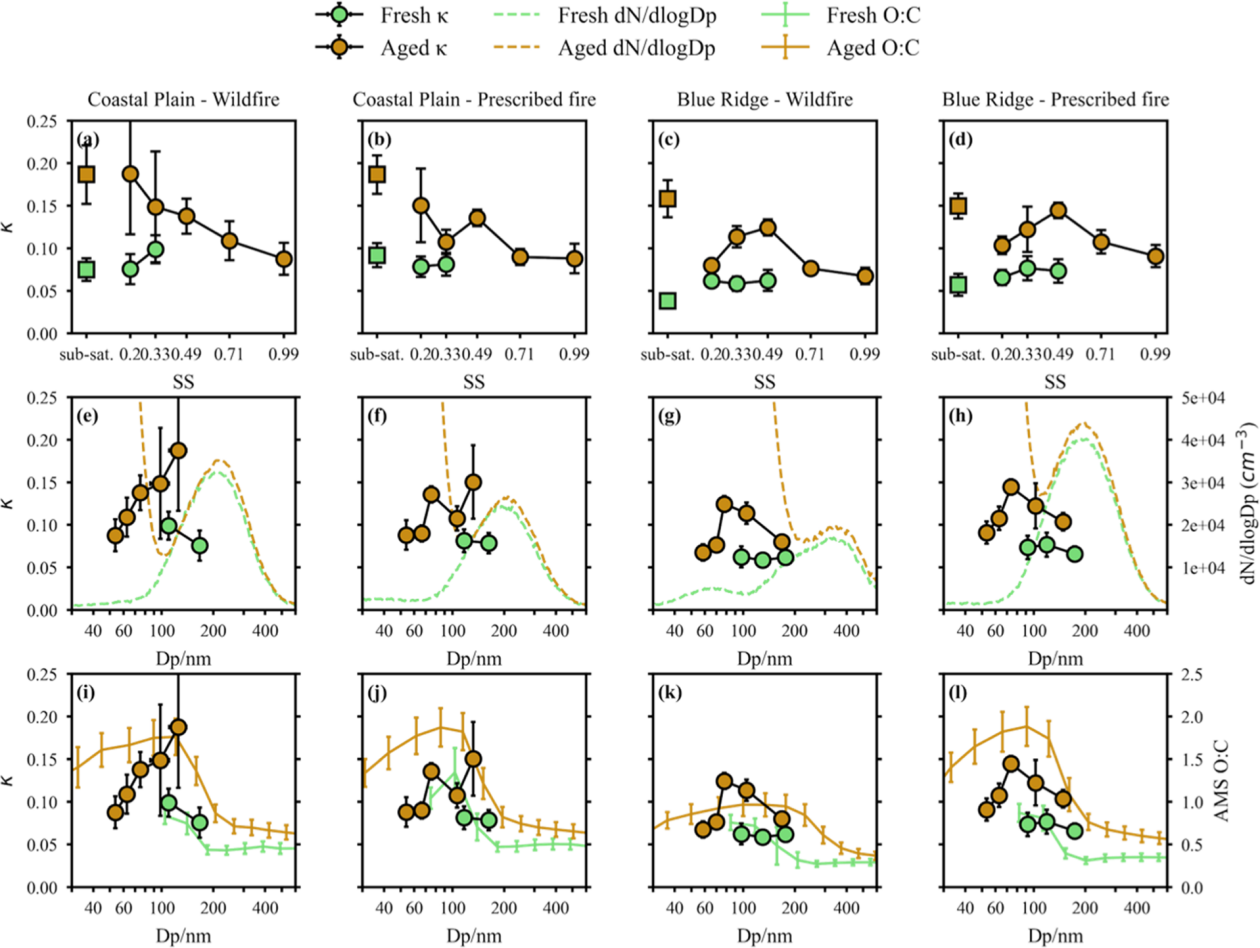
Comparison of CCN activity (κ_CCN_) across varying SS levels and with κ_QCM_ (panels a–d); κ_CCN_ as a function of activation diameters together with number size distributions (panels e–h); and κ_CCN_ as a function of activation diameters and the corresponding O/C ratios (panel i–l) for various BB particles.

Following photochemical aging, a nucleation mode emerged at small diameters as a result of homogeneous SOA formation from the oxidation of VOCs and subsequent nucleation in the PAM OFR (brown dashed lines in Figure 3e–h). Meanwhile, the number distributions of the original accumulation mode of BB particles showed minor changes, indicating that the influence of SOA on the accumulation-mode BB particles was insignificant. The photochemical ages of 3.2–7.4 days in the PAM OFR were atmospherically relevant, yet the short residence time of only a few minutes introduced kinetic differences. Rapid oxidation within the PAM OFR promoted homogeneous nucleation, and the high surface-area concentration of newly formed particles subsequently dominated vapor condensation, limiting condensational growth on accumulation-mode particles. The formation of nucleated particles from BB smoke, triggered by photochemical aging, has also been reported in chamber studies.^14,21^ Although the rapid nucleation process may not be fully representative of the evolution of size distributions under ambient aging conditions, it provided a unique opportunity to examine the hygroscopicity of the two modes separately.

Below, we refer to the fresh BB particles as POA and the nucleation-mode secondary particles as SOA. The physicochemical changes in the accumulation mode are attributed to the transformation of POA into OPOA. Specifically, minor changes (<50%) in particle number concentration were interpreted as indicative of OPOA formation, whereas the appearance of new particles accompanied by a >500% increase in number concentration was attributed to SOA formation, with mixed types falling in between (see Figure S8). The effects of photochemical aging on κ_CCN_ were evident but not uniform across different SS levels and particle diameters. At small activation diameters (<60 nm), the κ_CCN_ values of BB SOA (0.067–0.091) were comparable to those of fresh BB particles (0.061–0.087), indicating that SOA formation may not be an effective pathway for increasing the hygroscopicity of BB particles. At larger diameters, photochemical aging increased κ_CCN_ values to varying degrees. For BB particles from Coastal Plain burns, the κ_CCN_ values of OPOA (0.187 for wildfires and 0.150 for prescribed fires) were significantly higher than those of POA, despite minor changes in the size distributions. These results emphasize the role of heterogeneous oxidation of primary BB particles in altering their physicochemical composition through the formation of OPOA. For these particles, κ_QCM_ was also in good agreement with κ_CCN_ at around 130 nm (at the lowest SS in Figure 3a–d). In contrast, for aged particles from Blue Ridge burns, the κ_QCM_ values (0.158 for wildfires and 0.150 for prescribed fires) were considerably higher than the corresponding κ_CCN_ values at around 160 nm (0.080 for wildfires and 0.100 for prescribed fires). The observation that κ measured at subsaturation exceeds that measured at supersaturation can only be explained by higher hygroscopicity at larger diameters, attributable to size-dependent variations in chemical composition and mixing state. Fresh BB particles from Blue Ridge wildfires were unique in that the second mode was larger than that observed in the other burns, and subsequent aging produced a new particle mode extending to around 200 nm, which was the uppermost diameter captured by the CCN measurements. For aged Blue Ridge wildfire particles, the κ_CCN_ values were representative of the nucleated SOA, and the highest κ_CCN_ values were observed within the SOA mode. Since κ_QCM_ reflects the total particle mass and therefore primarily represents the OPOA mode, the divergence between the κ_CCN_ values measured at the largest diameter (SS = 0.20%) and the κ_QCM_ values for Blue Ridge particles can be attributed to the distinct physicochemical properties of SOA and OPOA.

Based on the measured κ_CCN_ across different particle-size modes and the observation that the bulk κ_QCM_ values exceeded κ_SOA_, we conclude that κ_OPOA_ is higher than κ_SOA_ for aged BB particles. Table 2 shows the κ_QCM_ values for fresh and aged BB particles as well as the mass ratios of SOA to OPOA after aging. Although most of the SOA mass enhancements were below 40%, κ_QCM_ increased by more than a factor of 2, suggesting that SOA formation was not a primary driver of the κ_QCM_ increases during aging.

**Table 2:** QCM-Derived κ Values for Fresh and Aged BB Particles and Corresponding SOA/OPOA Mass Ratios after Aging across Biomes and Fuel-Moisture Conditions

biomes	fuel moisture	κ_fresh_	κ_aged_	*m*_SOA_/*m*_OPOA_
Coastal Plain	wildfires	0.094 ± 0.025	0.187 ± 0.035	0.18
	prescribed fires	0.085 ± 0.015	0.187 ± 0.022	0.28
Blue Ridge	wildfires	0.067 ± 0.022	0.158 ± 0.02	0.79
	prescribed fires	0.082 ± 0.037	0.15 ± 0.015	0.36

Here, some κ_CCN_ values appeared lower than κ_QCM_ for aged BB particles, a discrepancy that can be explained only by the size dependence of κ_CCN_ and the influence of external mixing. In addition, no κ_QCM_ enhancement was observed as RH increased from 20% to 90%, which is distinct from the behaviors of inorganic salts and guaiacol NO_3_ oxidation SOA that exhibit deliquescence-like transitions.^50^ We therefore conclude that no discrepancy between subsaturation and supersaturation regimes was evident for the BB particles examined in this study.^12,19,33,34^

### 3.3 κ Dependence on O/C Ratio

We then examine the relationship between BBOA hygroscopicity and O/C ratios under the assumption that the BB particles investigated here were predominantly organic. Figure 3i–l shows the size-resolved O/C ratios measured by HR-ToF-AMS together with κ_CCN_. For fresh BB particles, the O/C ratio decreased by roughly a factor of 2 from 100 to 200 nm, suggesting that less-oxidized species preferentially condensed onto larger particles during primary emissions. However, the κ_CCN_ values remained nearly constant across this size range, indicating that the O/C ratio is not a reliable predictor of size-resolved κ_CCN_. For aged BB particles, much higher O/C ratios were observed for SOA (<100 nm) compared with OPOA (>200 nm), highlighting the distinct chemical compositions of these two modes. The transformation from POA to OPOA was also reflected by a notable increase in O/C ratios, although the changes in size distribution were minor. Overall, the trends in O/C ratios and κ_CCN_ were not well aligned, and no clear correlation could be established.

We further illustrate the relationship between κ_CCN_ and O/C ratios for POA, OPOA, SOA, and mixed types in Figure 4a. For SOA, we did not find any correlation between κ_CCN_ and O/C ratios, with all SOA κ_CCN_ values falling within 0.11 ± 0.05 (mean ± 2 × standard deviation). In comparison, the POA κ_CCN_ values were within 0.07 ± 0.02. When POA and larger-sized OPOA were considered together, their κ_CCN_ values displayed a strong correlation with O/C ratios (*R* ^2^ = 0.79), suggesting that heterogeneous oxidation can effectively increase κ_CCN_. This was further supported by the observation of an even stronger positive correlation (*R* ^2^ = 0.95) between κ_QCM_ and O/C ratios (Figure 4b). Note that the mass-averaged κ_QCM_ measurements were primarily influenced by larger-diameter OPOA particles, which were beyond the range of the CCN measurements. For bulk BB particles, we determined a regression of κ_QCM_ = (0.32 ± 0.02) × (O/C) – (0.05 ± 0.02), representing the first demonstration of a linear relationship between O/C ratio and hygroscopicity for both fresh and aged BBOA. The correlation slope for κ_CCN_ (0.08 ± 0.01; mean ± standard error) was lower than that for κ_QCM_, with κ_CCN_ exhibiting a broader spread in O/C ratios. This discrepancy may be explained for the following reasons. The CCNC-measured POA and OPOA between 100 and 150 nm corresponded to initially higher POA O/C ratios, resulting in elevated O/C values for both POA and OPOA within this size range. This reduced the apparent slope between O/C and κ_CCN_. In addition, the larger particles that dominated the κ_QCM_ values may exhibit a steeper dependence on O/C ratios. Therefore, κ_CCN_ for SOA, κ_CCN_ for POA + OPOA, and κ_QCM_ should be interpreted with consideration of their respective diameter ranges.

**Figure 4:**
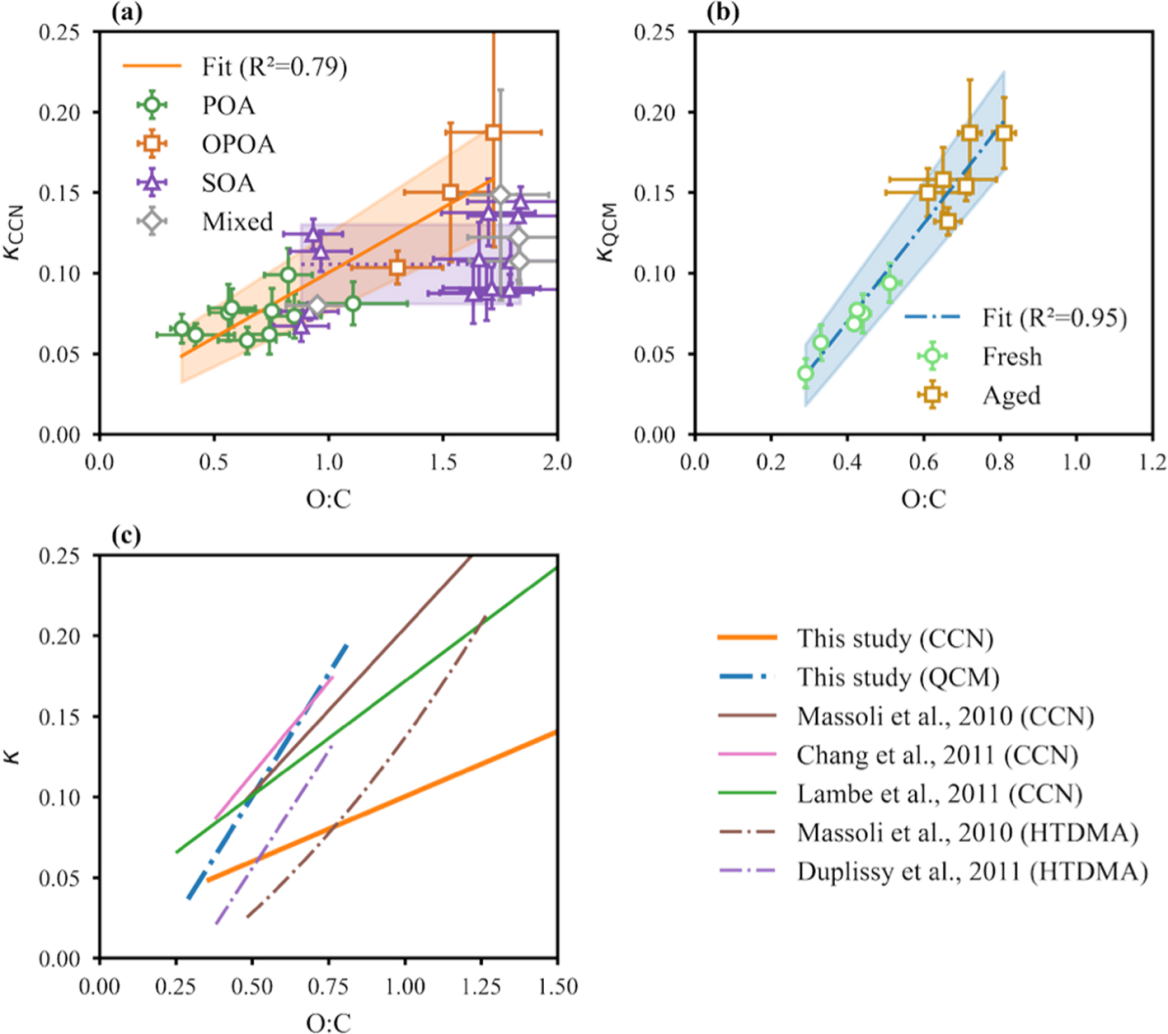
(a) Correlation between size-resolved CCN activity (κ_CCN_) and O/C ratios; (b) correlation between subsaturation hygroscopicity parameters (κ_QCM_) and O/C ratios; and (c) comparison of κ_OA_–O/C regression relationships across multiple studies. The gray shaded region in (a) denotes the uncertainty range of κ_CCN_ for SOA. The orange shaded region in (a) and the blue shaded region in (b) denote the uncertainty ranges of the respective regressions.

The reason for the positive relationship between OA hygroscopicity and O/C ratios has been the subject of extensive investigation.^31,35,51–54^ Several studies have suggested that the hygroscopicity parameter is strongly influenced by water solubility for single-compound OA^51,53^ or solubility-segregated OA.^35^ For single organic compounds, as the molecules become increasingly oxidized, they become more polar, generally leading to an increased water solubility. As the O/C ratio increases from near zero to high values, the properties of the organic compounds shift from insoluble to slightly soluble and eventually to highly soluble, corresponding to a general increase in κ.^51,53^ In the regime of high solubility, κ is no longer limited by solubility but is predominantly determined by the molecular weight of the organic compounds.^51,53,54^ For atmospheric OA comprising thousands of different species with variable solubility,^35,36,55^ the effect of solubility on hygroscopicity requires further validation. Wang et al.^31^ suggested that SOA hygroscopicity increases with O/C under supersaturated conditions because both variables are linked to molecular weight. Hygroscopicity is inversely related to molecular weight, and O/C ratio is also always negatively correlated with molecular weight because (1) smaller molecules need to be more oxidized to partition into the particle phase with sufficiently low volatilities and (2) fragmentation is an important mechanism leading to more oxidized OA. They found that essentially all organics were dissolved at the point of activation for the SOA they investigated, indicating that κ_SOA_ is not limited by solubility despite the presence of less-soluble species. The solubility limitation may be less important for complex OA mixtures that exhibit nonideality and multicomponent interactions. The lack of correlation between SOA κ_CCN_ and O/C ratios suggests that the highly oxidized BB SOA particles produced in the PAM OFR after 3.2 to 7.4 days of equivalent photochemical aging are already sufficiently soluble, and further oxidation does not enhance their hygroscopicity. Moreover, since these BB SOA particles were formed from similar organic vapors, their O/C ratios are not expected to correlate with molecular weight. For BB POA and OPOA, the trends in O/C ratios may be associated with variations in chemical species with different molecular weights. We also observed general consistency between subsaturation and supersaturation κ, as well as constant κ values across a wide RH range, supporting the conclusion that solubility limitation exerted minimal influence on the observed BBOA hygroscopicity. The observed positive relationship between BBOA κ and O/C ratios, primarily driven by the POA-to-OPOA transformation, may arise from relationships among compound molecular weight, O/C ratio, and volatility that governs gas-particle partitioning, as proposed by Wang et al.^31^

Figure 4c compares the κ–O/C regression lines for BBOA in this study with those from previous studies of laboratory SOA and ambient OOA.^27^ Chang et al.^32^ proposed a linear κ_org_–O/C relationship, κ_org_ = (0.23 ± 0.04) × (O/C), for O/C ratios between 0.4 and 0.8 based on CCN measurements at a rural site in Canada. Massoli et al.^30^ measured the hygroscopic growth and CCN activity of PAM OFR-generated SOA with O/C ratios ranging from 0.5 to 1.3. Applying a linear fit to κ_CCN_ yielded κ_org_ = (0.20 ± 0.02) × (O/C). Massoli et al. also fitted the hygroscopic growth factor at 90% RH, which was converted to a κ–O/C relationship and is plotted in Figure 4c. Duplissy et al.^28^ gathered HTDMA-derived κ values from various SOA and field-campaign OA, producing κ_org_ = 0.2 × (O/C) – 0.07. Lambe et al.^29^ generated SOA and oxidized APOA from 14 precursors in a PAM OFR, and most of the κ values measured by CCNC exhibited a linear relationship with O/C given by κ_org_ = (0.14 ± 0.03) × (O/C) + 0.02, except for very hydrophobic APOA with O/C < 0.25. We note that all of the equations have been adjusted from their original forms because O/C ratios were scaled by a factor of 1.27 to account for differences in O/C calculation methods.^56^

The relationship between κ_QCM_ and O/C ratios for BBOA subsaturation hygroscopicity is comparable to that between κ_CCN_ and O/C for SOA and ambient OOA, consistent with the agreement in BB particles κ between subsaturation and supersaturation regimes. In comparison, at lower O/C ratios, the κ_HTDMA_ of SOA measured at high RH is significantly lower than the κ_CCN_ measured at supersaturation.^28–30^ This discrepancy can be explained by the nonideal mixing of hydrophobic and hydrophilic organic compounds with water, which suppresses κ at the high RH levels typically applied in HTDMA measurements.^37^ Such nonideal mixing and gradual dissolution mechanisms^35,36^ were insignificant for the BBOA investigated here.

## 4 Discussion and Implications

This study investigated the hygroscopicity and CCN activity of organic-dominant BB particles across a wide range of RH (20–90%) using QCM hygroscopicity measurements and SS (0.13–0.99%) using size-resolved CCN activity assessments. Our results revealed continuous water absorption without deliquescence for both fresh and aged BB particles, similar to the behavior of SOA. The derived κ_QCM_ values for fresh BB particles ranged from 0.04 to 0.10 and notably increased to 0.14–0.20 after photochemical aging. These results indicate that BB organic particles can contribute to the aerosol liquid water content over a broad humidity range and participate in cloud processes during atmospheric transport. We examined the effects of fuel-bed types from different ecoregions and fuel moistures, which caused smaller variations in BB particle hygroscopicity compared to photochemical aging. However, additional laboratory simulations using fuel beds representative of a wider range of ecoregions are warranted to further evaluate the generality of this conclusion.

The hygroscopicity and chemical composition measurements together revealed substantial chemical heterogeneity in the investigated BB particles, as indicated by the size-dependent O/C ratios and the distinct chemical compositions of SOA and OPOA modes after aging. Specifically, highly oxidized SOA in the nucleation mode exhibited relatively low κ values compared to the profoundly elevated κ values for OPOA formed through heterogeneous aging, consistent with a previous study.^20^ We also found that κ_SOA_ showed no correlation with O/C ratios, whereas the κ_CCN_ values of POA and OPOA together exhibited a strong positive relationship with O/C ratios. These findings highlight the importance of distinguishing the roles of different aging pathways in the atmospheric evolution of BB particles and underscore the potential pitfalls of directly linking the changes in physical properties, such as size-dependent κ_CCN_, to chemical composition transformations in highly heterogeneous particles.

We conclude that the subsaturated and supersaturated κ values for the investigated BB particles are consistent after accounting for chemical heterogeneity and size-dependent CCN activity.^15,19,34^ As a caveat, hygroscopic growth data at very high RH (>90%) were not available in this study. Establishing a more robust linkage between subsaturated and supersaturated κ will require future investigations that include more comprehensive measurements.

We demonstrated a linear relationship between O/C ratios and κ_QCM_ by combining fresh and aged BBOA. The regression result aligns with previous findings for laboratory SOA and ambient OOA^28–30,32^ and may help refine a generalized parameterization inclusive of both SOA and BBOA with acceptable uncertainty. However, further research is still needed to validate these relationships and to fully understand the influence of solubility, molecular weight, and photochemical oxidation on the hygroscopic properties of BB aerosols. In addition, HR-ToF-AMS may have missed some compositions of BB particles, including black carbon (BC) and refractory organics. Since BC accounted for less than 10% of the total particle mass,^39^ a fraction of the organics could also have been missed by HR-ToF-AMS due to their BC-like property. These compositions may include dark brown carbon or tar-balls, as described in previous studies.^57,58^ The varying relative fractions of nonrefractory and refractory organics and their influence on the overall properties of BB particles from different fuel beds and burning conditions should receive greater attention in future investigations.

## Supplementary Material



## Notes

Tabulated experimental conditions; aerosol particle characteristics; sample descriptions; and typical experimental profiles, method validation, and raw experimental results (PDF)
