# Supplementary material for: Hygroscopicity and Cloud Condensation Nuclei Activity of Fresh and Aged Biomass Burning Particles
Source: ACS EST Air. 2026 Feb 13;3(3):697–709. doi: 10.1021/acsestair.5c00331 (PMC12993806; doi:10.1021/acsestair.5c00331)
Supplement: Supplementary file 1 [file ea5c00331_si_001.pdf]

Supplementary Information

Hygroscopicity and cloud condensation nuclei activity of fresh and aged biomass burning particles

Bin Bai [a, 1], Aishwarya Singh [b, c, 1], Tianchang Xu [d], Christos Stamatis [e], Kezhou Lu [a, f], Nara Shin [a, g], Chase K. Glenn [h, i], Omar El Hajj [h], Kruthika V. Kumar [h], Anita Anosike [h], Muhammad Isa Abdurrahman [h], Sachin S. Gunthe [b, c], Joseph J. O'Brien [j], Gabriel Isaacman-VanWertz [e], Rawad Saleh [h], Nga L. Ng [a, d, k], Pengfei Liu [a*]

[a] School of Earth and Atmospheric Sciences, Georgia Institute of Technology, Atlanta, Georgia 30332, USA

[b] EE Division, Department of Civil Engineering, Indian Institute of Technology Madras, Chennai 600036, India

[c] Centre for Atmospheric and Climate Sciences, Indian Institute of Technology Madras, Chennai 600036, India

[d] School of Chemical and Biomolecular Engineering, Georgia Institute of Technology, Atlanta, Georgia 30332, United States

[e] Department of Civil and Environmental Engineering, Virginia Tech, Blacksburg, Virginia 24061, United States

[f] Department of Atmospheric and Oceanic Sciences, University of California, Los Angeles, California 90095, United States

[g] School of Health Sciences, Purdue University, West Lafayette, Indiana 47907, USA

[h] School of Environmental, Civil, Agricultural, and Mechanical Engineering, University of Georgia, Athens, Georgia 30602, USA

[i] Aerodyne Research, Billerica, Massachusetts 01821, United States

[j] USDA Forest Service Southern Research Station, Athens, Georgia 30602, United States

[k] School of Civil and Environmental Engineering, Georgia Institute of Technology, Atlanta, Georgia 30332, United States

[1] B.B. and A.S. contributed equally to this work

[*] *E-mail: pengfei.liu@eas.gatech.edu*

Submitted: Nov 2025

Contents of this file:

Table S1-4

Figure S1-8

Table S1. Photochemical age (PCA), O:C ratio changes, and κ_{QCM} changes measured in the PAM OFR for each burn.

Burn Type	Average PCA (1st aging period)	Average PCA (2nd aging period)	ΔO:C	$\Delta\kappa_{QCM}$
Coastal Plain - Wildfire	5.7	5.4	0.28 ± 0.04	0.11 ± 0.04
Coastal Plain - Prescribed fire	5.8	5.3	0.30 ± 0.08	0.09 ± 0.03
Blue Ridge - Wildfire	3.2	3.3	0.36 ± 0.12	0.12 ± 0.02
Blue Ridge - Prescribed fire	5.4	5.9	0.33 ± 0.13	0.09 ± 0.02
Piedmont - Wildfire	5.1	5.1	0.29 ± 0.01	0.08 ± 0.01
Piedmont - Prescribed fire	5.0	5.8	0.25 ± 0.04	0.06 ± 0.01

Table S2. Mass loadings and collection efficiencies of QCM-sensor samples for BB particles.

Eco-region	Burn	Age	Run No.	Mass loading/μg	Collection efficiency	Sensor Type
Coastal Plain	Wildfire	Fresh	1	8.9	0.38	SiO_2-coated
			2	8.0	0.18	Gold-coated
			3	18.8	0.48	SiO_2-coated
		Aged	1	2.8	0.10	SiO_2-coated
			2	4.2	0.20	Gold-coated
	Prescribed fire	Fresh	1	6.3	0.55	SiO_2-coated
			2	4.2	0.40	Gold-coated
			3	10.3	0.37	Gold-coated
			4	5.5	0.50	Gold-coated
		Aged	1	4.7	0.21	Gold-coated
			2	3.3	0.25	SiO_2-coated
			3	7.3	0.21	SiO_2-coated
Blue Ridge	Wildfire	Fresh	1	4.3	0.21	Gold-coated
			2	23.4	0.46	SiO_2-coated
			3	6.6	0.14	Gold-coated
		Aged	1	9.9	0.22	Gold-coated
			2	15.1	0.13	SiO_2-coated
			3	7.7	0.45	Gold-coated

	Prescribed fire	Fresh	1	3.4	0.20	Gold-coated
			2	6.6	0.32	SiO_2-coated
			3	13.6	0.35	SiO_2-coated
		Aged	1	7.2	0.30	Gold-coated
			2	11.6	0.31	Gold-coated
Piedmont	Wildfire	Fresh	1	17.9	0.43	SiO_2-coated
			2	13.1	0.39	SiO_2-coated
		Aged	1	2.4	0.38	SiO_2-coated
			2	4.0	0.26	SiO_2-coated
	Prescribed fire	Fresh	1	37.9	NA	SiO_2-coated
			2	12.6	NA	Gold-coated
			3	19.7	NA	SiO_2-coated
			4	9.7	NA	Gold-coated
		Aged	1	7.3	NA	SiO_2-coated
			2	3.1	NA	Gold-coated
			3	2.5	NA	Gold-coated

Table S3. Density, organic mass fraction, O:C ratio, and average κ_{QCM} value for different BB particle types.

Ecoregion	Burn	Age	density	f_{org}	O:C	κ_{QCM}
Coastal Plain	Wildfire	Fresh	1.39 ± 0.04	0.97	0.44 ± 0.02	0.075 ± 0.012
		Aged	1.61 ± 0.03	0.97	0.72 ± 0.03	0.187 ± 0.033
	Prescribed fire	Fresh	1.49 ± 0.03	0.97	0.51 ± 0.03	0.094 ± 0.012
		Aged	1.62 ± 0.02	0.97	0.81 ± 0.03	0.187 ± 0.022
Blue Ridge	Wildfire	Fresh	1.32 ± 0.03	0.97	0.29 ± 0.01	0.038 ± 0.009
		Aged	1.32 ± 0.06	0.98	0.65 ± 0.14	0.158 ± 0.02
	Prescribed fire	Fresh	1.37 ± 0.02	0.97	0.33 ± 0.01	0.057 ± 0.011
		Aged	1.50 ± 0.11	0.98	0.61 ± 0.11	0.15 ± 0.015

Table S4. Fraction of more-hydrophilic and less-hydrophilic particle populations and the corresponding κ_{QCM} value derived at SS = 0.2% for different BB particle types.

Ecoregion	Burn	Age	More-hydrophilic fraction	κ_{CCN} (more-hydrophilic)	Less-hydrophilic fraction	κ_{CCN} (less-hydrophilic)
Coastal plain	Wildfires	Fresh	0.27	0.20 ± 0.04	0.73	0.04 ± 0.02
		Aged	0.53	0.28 ± 0.07	0.47	0.04 ± 0.02
	Prescribed fire	Fresh	0.34	0.16 ± 0.05	0.66	0.04 ± 0.01
		Aged	0.56	0.15 ± 0.04	0.44	0.05 ± 0.02
Blueridge	Wildfire	Fresh	0.34	0.14 ± 0.03	0.66	0.03 ± 0.01
		Aged	0.38	0.15 ± 0.04	0.61	0.05 ± 0.02
	Prescribed fire	Fresh	0.22	0.16 ± 0.03	0.78	0.04 ± 0.02
		Aged	0.38	0.19 ± 0.05	0.61	0.05 ± 0.02

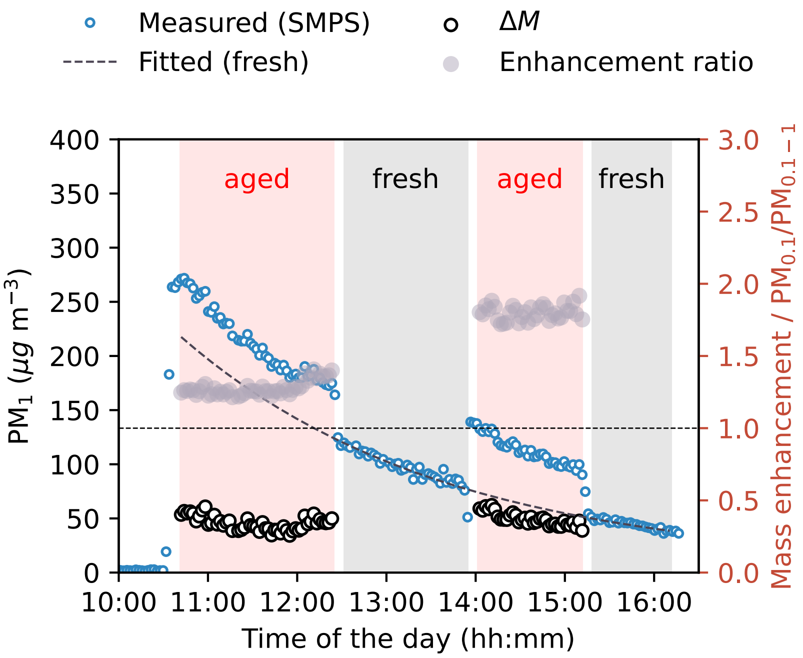

Figure S1. Temporal profile of BB particle mass concentrations measured by SMPS with a typical aged–fresh–aged–fresh sequence.

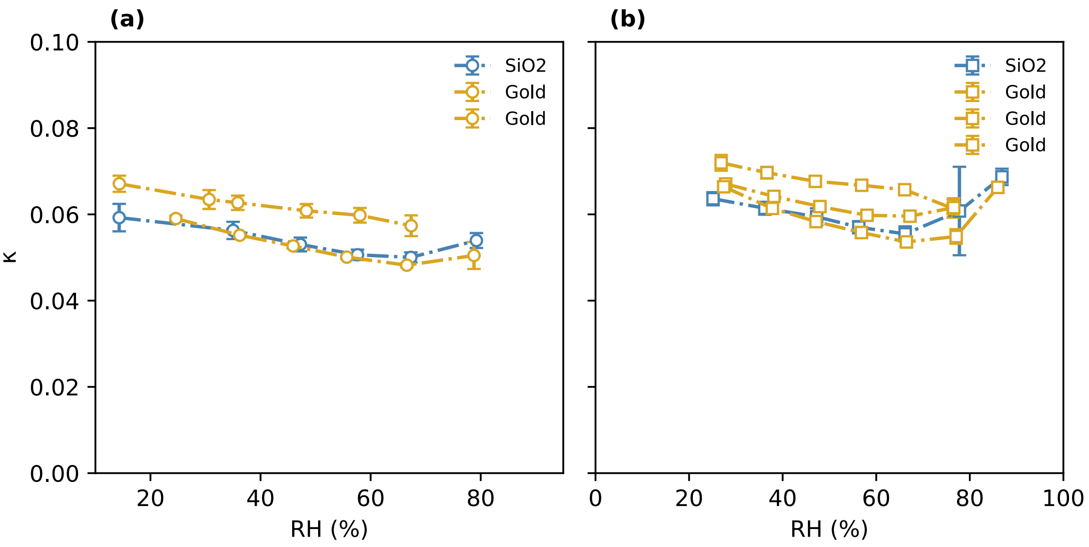

Figure S2. Comparison of hygroscopicity (κ_{QCM}) derived from different QCM substrates for fresh BB particles from (a) Coastal Plain wildfire and (b) Coastal Plain prescribed fire.

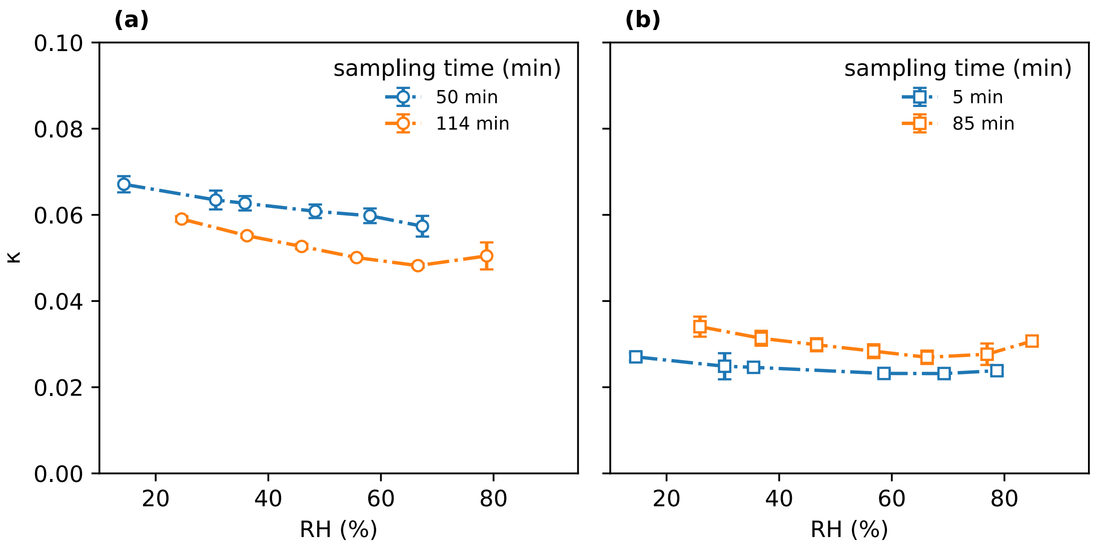

Figure S3. Comparison of hygroscopicity (κ_{QCM}) for different sampling times for fresh BB particles from (a) Coastal Plain wildfire and (b) Blue Ridge wildfire.

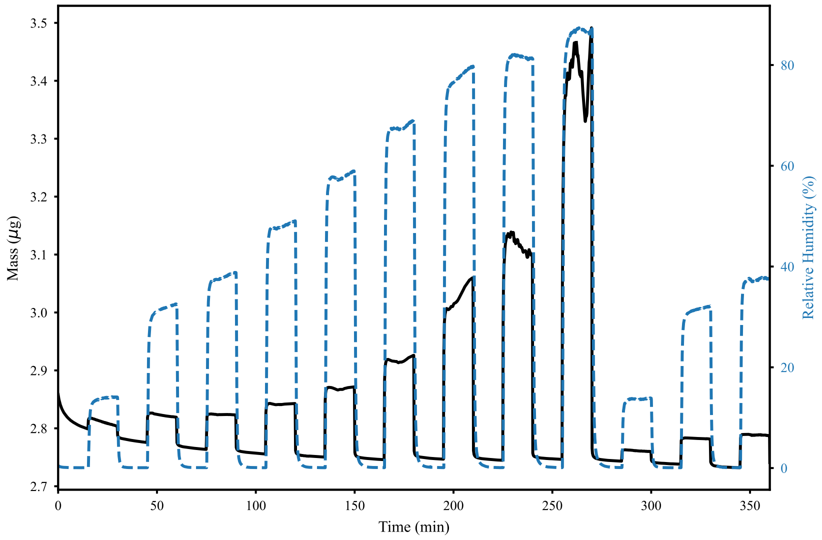

Figure S4. Particle and absorbed water mass on a QCM sensor and the corresponding RH profile during a typical hygroscopicity measurement experiment.

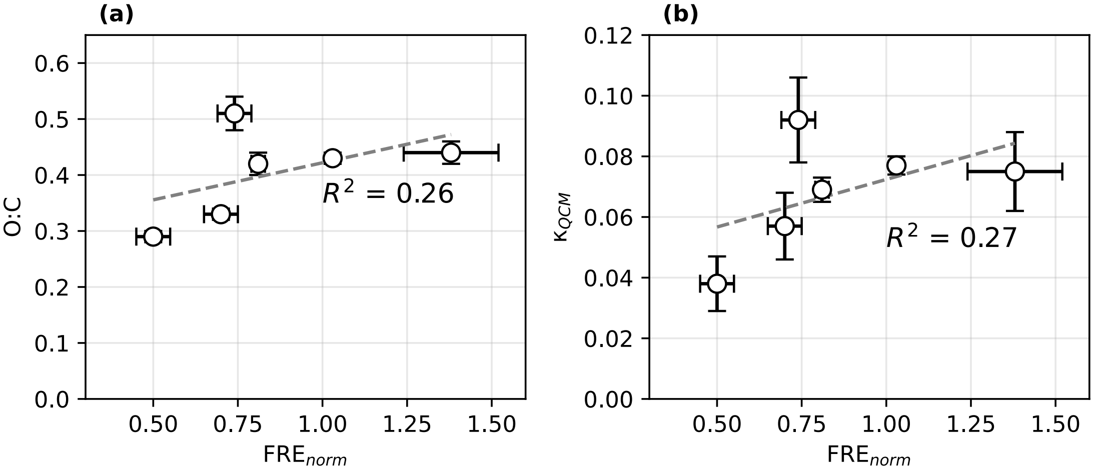

Figure S5. Correlation between average fire radiative energy normalized by the available fuel mass loading (FRE_{norm}) and (a) O:C ratios and (b) hygroscopicity (κ_{QCM}) of fresh BB particles across different burns.

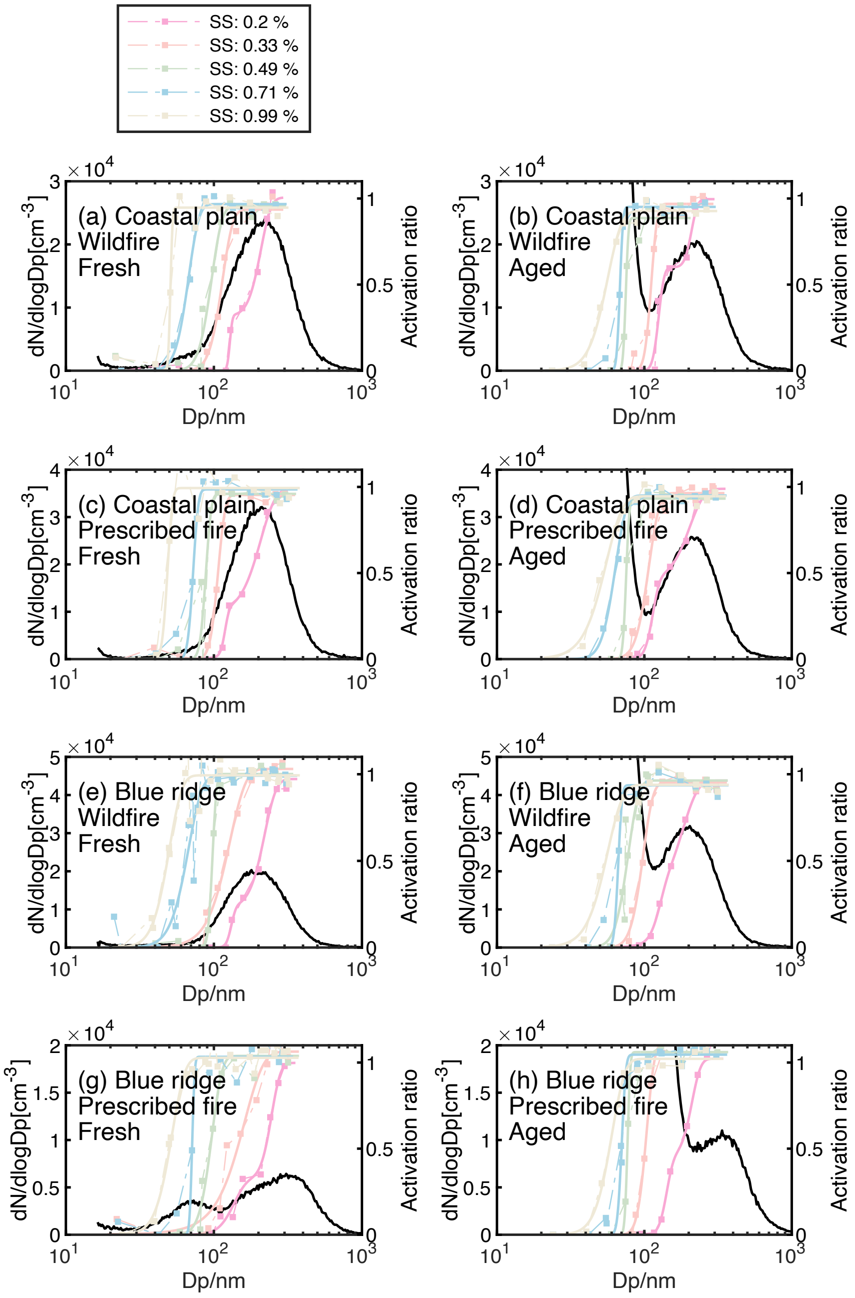

Figure S6. CCN activation curves and corresponding particle number size distributions for different BB particle types.

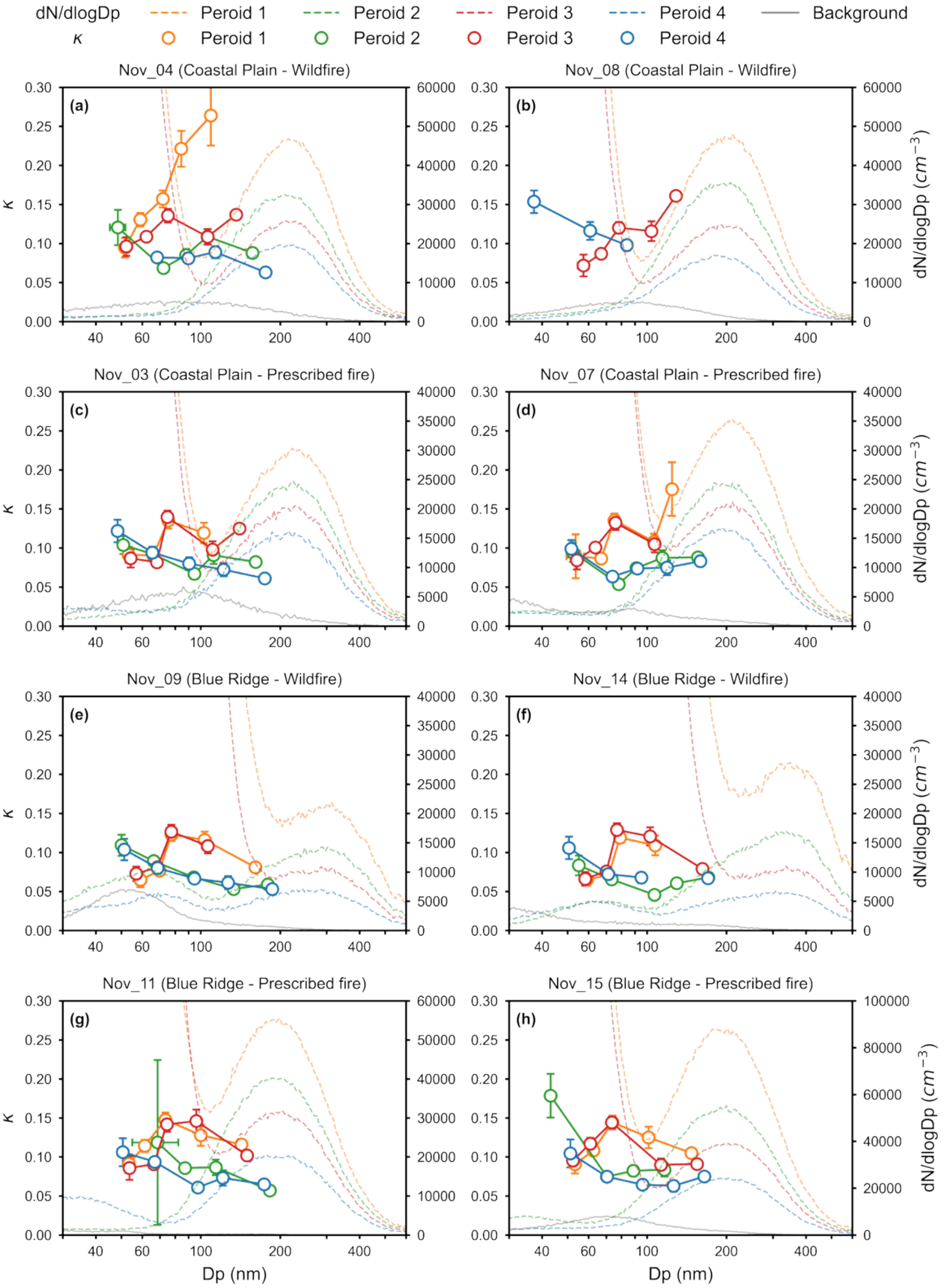

Figure S7. CCN activity as a function of activation diameter, shown together with particle number size distributions during different experimental periods. Periods 1 and 3 correspond to aged particles, while periods 2 and 4 correspond to fresh particles.

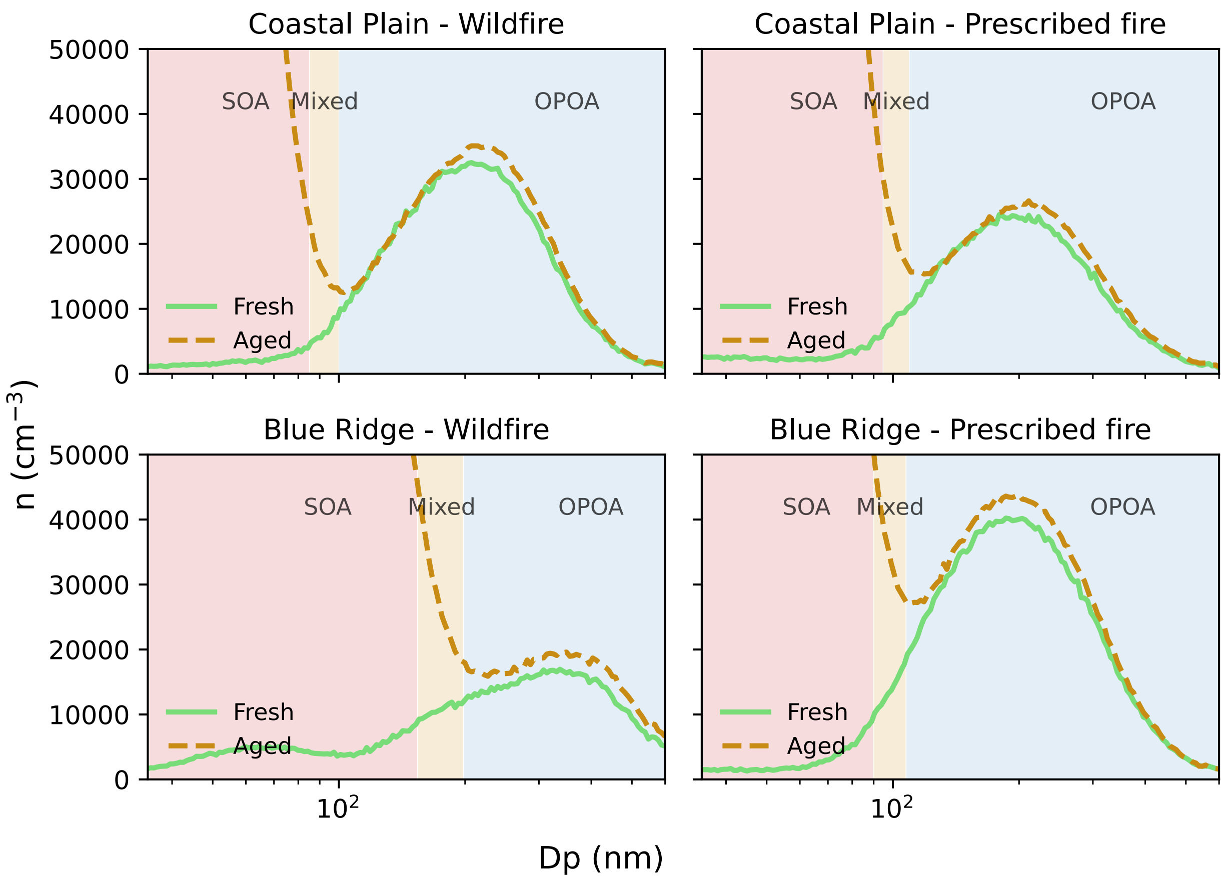

Figure S8. Size ranges for SOA, OPOA, and mixed particles for aged BB particles, classified based on changes in particle number concentrations during photochemical aging.
